# *Drosophila* in the Heart of Understanding Cardiac Diseases: Modeling Channelopathies and Cardiomyopathies in the Fruitfly

**DOI:** 10.3390/jcdd3010007

**Published:** 2016-02-18

**Authors:** Ouarda Taghli-Lamallem, Emilie Plantié, Krzysztof Jagla

**Affiliations:** GReD (Genetics, Reproduction and Development laboratory), INSERM U1103, CNRS UMR6293, University of Clermont-Ferrand, 28 place Henri-Dunant, 63000 Clermont-Ferrand, France; ouarda.taghli-lamallem@udamail.fr (O.T.-L.); emilie.plantie@udamail.fr (E.P.)

**Keywords:** human cardiac disease, *Drosophila*, channelopathies, cardiomyopathies, sarcomeric proteins, cytoskeletal proteins, K^+^ channels

## Abstract

Cardiovascular diseases and, among them, channelopathies and cardiomyopathies are a major cause of death worldwide. The molecular and genetic defects underlying these cardiac disorders are complex, leading to a large range of structural and functional heart phenotypes. Identification of molecular and functional mechanisms disrupted by mutations causing channelopathies and cardiomyopathies is essential to understanding the link between an altered gene and clinical phenotype. The development of animal models has been proven to be efficient for functional studies in channelopathies and cardiomyopathies. In particular, the *Drosophila* model has been largely applied for deciphering the molecular and cellular pathways affected in these inherited cardiac disorders and for identifying their genetic modifiers. Here we review the utility and the main contributions of the fruitfly models for the better understanding of channelopathies and cardiomyopathies. We also discuss the investigated pathological mechanisms and the discoveries of evolutionarily conserved pathways which reinforce the value of *Drosophila* in modeling human cardiac diseases.

## 1. Introduction

Cardiovascular diseases (CVDs) remain the leading cause of death worldwide, with over four million deaths per year in Europe (46% of all deaths) and 787,650 in the United States (31.9% of all deaths) [1,2]. The CVDs affect the structure and/or function of the heart and blood vessels and comprise congenital, coronary and rheumatic heart diseases, hypertrophic, dilated and restrictive cardiomyopathies, as well as cardiac arrhythmias and stroke. The molecular and genetic defects underlying congenital heart diseases and cardiomyopathies are still not fully understood, awaiting further investigations to identify affected genes and pathways.

Cardiomyopathies are defined as myocardial disorders in which the cardiac dysfunction ranges from symptomless to major health complications such as arrhythmia, heart failure and sudden cardiac death. Cardiomyopathies are classified, based on ventricular morphology and function, into hypertrophic (HCM), dilated (DCM), arrhythmogenic right ventricular (ARVC), and restrictive cardiomyopathy (RCM) [3]. They can be caused by monogenic mutations inherited in Mendelian fashion, and are thus called inherited cardiomyopathies.

HCM, the most common with an estimated prevalence of one in 500 individuals [4], is characterized by increased left ventricular (LV) wall thickness, myocyte hypertrophy, myofibrillar disarray, increased fibrosis and impaired LV diastolic function [5]. DCM is characterized by dilation and impaired contraction of the left or both ventricles (systolic dysfunction) which leads to progressive heart failure and sudden cardiac death from ventricular arrhythmia [6]. The prevalence of DCM is estimated to be one in 2500 [7]. RCM is an uncommon form of cardiomyopathy, characterized by increased stiffness and restrictive filling of the left and/or right ventricle despite normal wall thickness and systolic function [8]. RCM is rare and several reports suggest genetic overlap with HCM [9,10]. ARVC has a prevalence of at least one in 1000 and corresponds to a chronic and progressive myocardial disorder, leading to sudden cardiac death in general in people less than 35 years old [11,12]. Early ARVC bears more resemblance to channelopathies (ion channel diseases) such as Long QT and Brugada syndrome [13] and as the disease progresses, typical histomorphological changes occur including myocyte loss, fibrosis and adiposis [14].

Genetically engineered animal models of cardiovascular diseases provide valuable tools to investigate the molecular and cellular mechanisms of CVD pathogenesis and to evaluate therapeutic strategies (reviewed in [15,16,17]). The fruitfly exhibits strong gene conservation with human genes, including 75% of disease-related genes, and has proven to be an accurate model for studying human diseases ranging from neurological and endocrine diseases to muscular and cardiac disorders [18,19,20,21,22,23].

Following the discovery of the homeobox transcription factor Tinman (Tin) in *Drosophila* [24], regulatory cardiogenic network and conserved mechanisms with higher organisms have been elucidated [25,26]. In particular, it has been shown that the cardiac master genes *tinman*/*Nkx2-5*, *neuromancer*/*Tbx20*, *pannier*/*GATA4/6* and *dHand*/*Hand* not only specify the heart during development but also play a role in heart function in both the adult fly and in humans [27,28,29,30,31]. As a consequence, the extended conservation of transcriptional and signaling networks that control cardiac development and heart physiology made the fly a model of choice for studying human cardiac diseases [19,25,32,33,34,35,36].

**Figure 1:**
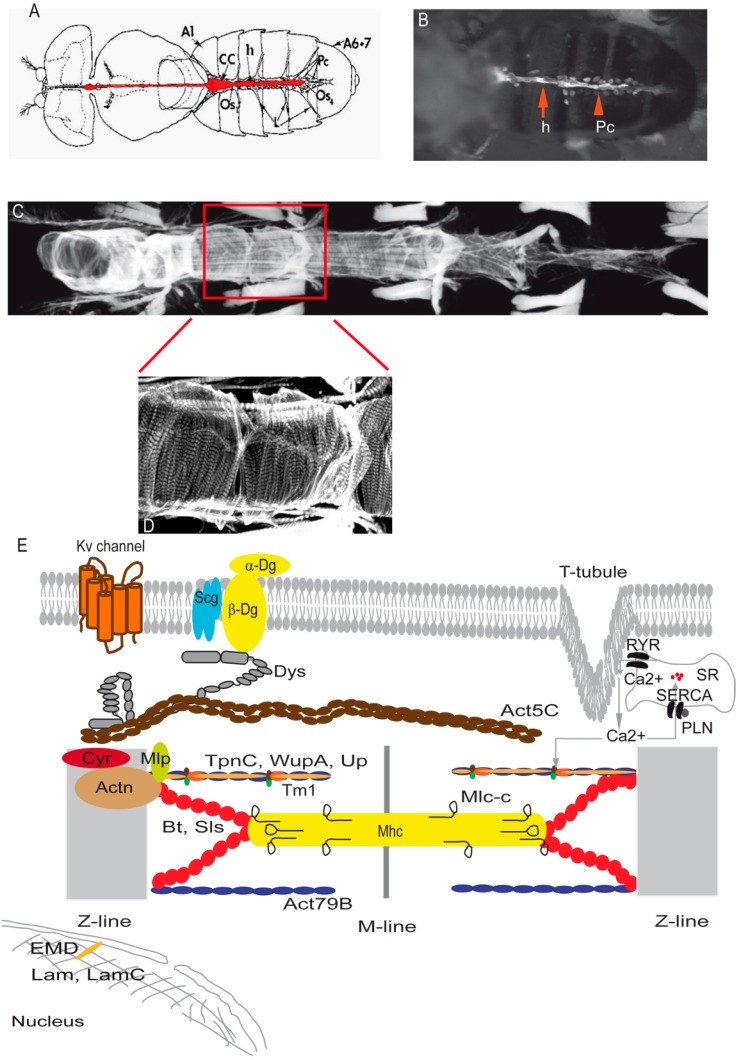
Adult heart structure and schematic representation of the cytoarchitectural proteins involved in cardiomyopathies. (**A**) Illustration demonstrating the heart tube located along the dorsal abdominal midline. CC: conical chamber; Os: ostia; h: heart; Pc: pericardial cells; A1: Abdominal segment 1; (**B**) Semi-intact preparation of *Drosophila* with ventral abdominal cuticle showing the green fluorescent protein expression in the heart tube (Hand-Gal4 > GFP); (**C**) Representative confocal stacks of the fly heart (anterior to left) stained with actin-phalloidin. Abdominal segment 2 is outlined in red; (**D**) Representative confocal stacks of A2 segment stained with actin-phalloidin revealing detail of heart structure; (**E**) Schematic representation of the cytoarchitectural components in flies. Some of the proteins studied induced cardiomyopathies in *Drosophila.* Kv channel: voltage-activated potassium channel; Dg: Dystroglycan; Scg: Sarcoglycan; RYR: ryanodine receptors; SERCA: Sarcoendoplasmic reticulum Ca^2+^ ATPase; SR: sarcoplasmic reticulum; PLN: Phospholamban; Dys: Dystrophin; Act: Actin; Cyr: Cypher; Actn: α-actinin; Mlp: Muscle LIM protein; Bt: Bent; Sls: Sallimus; TpnC: Troponin-C; WupA: Troponin-I ; Up: Troponin-T; Tm1: tropomyosin; Mhc: Myosin heavy chain; Mlc: Myosin light chain; EMD: Emerin; Lam: Lamin.

The *Drosophila* heart is a relatively simple linear tube located dorsally in the body (Figure 1A,B). It originates, like the vertebrate heart, from the lateral part of the mesoderm. The fly heart comprises two rows of contractile cells that form an inner lumen known as the myocardium and the non-muscular pericardial cells that align along the myocardial cells. The contractile cells contain spirally oriented myofibrils covered by a ventral layer of longitudinal non-myocardial muscle cells running along the heart tube (Figure 1C,D). Importantly, *Drosophila* cardiomyocytes have a sarcomeric structure and components similar to mammalian cardiac cells [37] (Figure 1E).

The availability of genetic tools makes the fruitfly not only an excellent model for investigating heart development but also for studying heart function and cardiac aging. The development of a large set of cardiac physiology assays to measure heart rate, rhythmicity, and contractility provided means to assess heart function defects in the generated fruitfly models of cardiac diseases [22]. Among them, extracellular and intracellular electrical recordings have been developed to monitor the fly electrocardiogram (ECG) [38,39,40]. Intracellular action potentials have been reported from both larvae and intact pupae. Action potentials were recorded from the posterior part of larval heart preparations with a standard microelectrode recording technique [40]. This study has identified that the two-pore domain potassium channel Ork1 likely sets the resting membrane potential and regulates heart rate and rhythm. In pupae, the intracellular recording was performed by sharpened tungsten electrodes that were inserted near the anterior part and in the caudal end of the heart [38]. The ECG generated from pupae bearing the shibire mutation showed irregular and compromised electrical currents, with a slower and less rhythmic heartbeat [38]. The extracellular recording system consists of using a micropipette tip to monitor the potential generated by spontaneous heart contractions for a 6–8 h period. An excitatory effect has been demonstrated while exposing the heart to octopamine with a reduction in amplitude of the extracellular potential [39]. Optical recording of cardiac activity directly through the cuticle [41] and projecting infrared light through the dorsal side of the abdomen [42] allowed recording pulsations of the heart. The noninvasive optical detections based on hearts expressing Green Fluorescent Protein (GFP) allowed observation under the microscope of the heart movements through the dorsal cuticle [41]. However, the projection of infrared light through the dorsal side of the abdomen followed by the collection of the pulse signal by a multichannel sensor chip has proven effective for obtaining a survey of periodic heartbeat reversals [42]. Similarly, the heart movements were tracked by a photodiode-transistor-based assay and video microscopy [43,44] whereas in live pupae, an edge-tracing system has been developed for tracking heart wall movements [45]. Measurements of transmitted light through the late-stage larvae change in consonance with the heart movements as the heart changes its shape in a diastole/systole cyclical fashion. The lightening and the darkening of the heart wall thus produce a trace of changing light exposure reflecting the cardiac rate and rhythmicity [43,44]. A similar method has been employed in which pixel-tracing camera technology has replaced the photodiode [45]. To assess heart failure (cardiac arrest or fibrillation) under stress conditions, a method that employs external electrical pacing or elevated temperature to drive increased heart rates has been useful in gauging cardiac performance [45,46,47]. In such assays, adult hearts are paced to a higher rate by increasing the temperature [46] or by passing through a current of 6 Hz for a time length of 30 s [45]. Immediately after pacing, the flies are scored depending on whether the heart can still contract following the stimulation or whether it fails to beat. Both cardiac stress tests were used to compare the effects of different mutations and the effect of age on heart performance. Moreover, the so-called Semi-automatic Optical Heartbeat Analysis (SOHA) method based on high-speed movies recorded from semi-intact adult fly heart preparations allowed detailed image-based analysis and characterization of cardiac parameters [48,49] whereas the optical coherence tomography (OCT) has been successfully adapted to assess cardiac parameters in awake flies [50]. The SOHA method relies on using two algorithms that have been developed to combine information extracted from overall darkness changes of the frame movie and pixel-wide intensity changes detected only in the regions that are moving from one frame to another in recorded images at a speed of 150 frames per second. The noninvasive image acquisition technique OCT has also been adapted to flies and permits detailed measurements of live cardiac performance in intact animals. In this technique, the internal space of the heart chamber is detected in real time as it shortens and expands through an ultrasound exposure. Both methods allow us to produce high-resolution qualitative records of heart wall movements (M-mode) and detailed parameters of cardiac function including diastolic/systolic interval diameters, fractional shortening, and heart rate. More recently, an alternative cardiac-specific pacing approach based on optogenetics has been developed to investigate rhythm disorders [51]. This method combines transgenic flies expressing channelrhodopsin-2 (ChR2), a light-gated cation channel, in the heart, and optical coherence microscopy to noninvasively analyze the heart structure and function *in vivo* [51]. Optogenetic pacing and capture of the response of the ChR2-expressing *Drosophila* heart to stimulation pulses can be performed at different developmental stages, including larva, pupa and adult. Finally, an atomic force microscopy–based indentation approach was applied to measure the myocardial stiffness, offering a mean to investigate heart cells’ mechanical properties and diastolic dysfunction [52]. Briefly, this technique uses a silicon cantilever with a 2 µm borosilicate sphere tip that is placed directly on the heart, stepped down and its deflection measured with a laser, providing a relative measure of stiffness. Using this approach, it has been shown that the stiffness of the heart changes with age or due to a particular genetic background.

In this review, we provide an overview of the fly model contributions to the understanding of genetic determinants and cellular pathways underlying channelopathies and cardiomyopathies. We discuss examples of insights gained from *Drosophila* models into pathogenic mechanisms involved in the cardiac remodeling processes or dysfunction which contribute to heart failure. We also address the challenges in CVD modeling in terms of therapy development, modifier screens and polygenic disorder investigations. Overall, this review pinpoints the growing interest in the fruitfly model and its applications to cardiac disorders.

## 2. Cardiovascular Disease Modeling Using *Drosophila*

With the recently developed techniques to assess *Drosophila* heart function, this model has been used to study channelopathies [40,44,48,53,54] and inherited cardiomyopathies including DCM, RCM and HCM [55,56,57,58,59,60].

### 2.1. Channelopathies

Ion channels are pore-forming proteins that selectively control ionic movement across the cell membrane and coordinate electrical signals in most tissues. Ion channels are either depolarizing cells, by moving positively charged ions in, or repolarizing cells, by moving positively charged ions out. Mutations in genes coding for ion channel subunits or any related regulators cause human diseases known as channelopathies, including Brugada syndrome and Long QT syndrome. We focus here only on the heart channelopathies.

*Drosophila* has made an important contribution in the field of channels, thanks to the forward genetic approach, by the identification and cloning of the *Shaker*, *ether-a-go-go*, *seizure*, and *slowpoke* genes. Genetic screens conducted in flies first allowed us to identify the voltage-activated potassium channel gene (*K_v_*) named *Shaker*, a member of the *K_v_1* family [61,62,63]. One member of this family in humans, channel *K_v_1.5*, has been involved in cardiac repolarization and associated with atrial fibrillation and is a target of anti-arrhythmic drugs [64,65]. The screening for shaker-related genes in flies allowed the discovery of other K^+^ channels such as *Shab*, *Shaw*, and *Shal* [66,67,68] and subsequent cloning of their orthologs in mammals, *K_v_2.1*, *K_v_3*, and *K_v_4.3*, respectively [69]. In *Drosophila*, blocking the *Shab* channel resulted in the slowing of heart beats but, unlike other K^+^ channels, did not lead to cardiac arrhythmia [54] (Table 1). In vertebrates, *Kv2.1* was found enriched in human ventricles and in mice and plays a role in repolarizing current in ventricular myocytes [70,71]. Also, the expression of the *K_v_4.3* channel is altered in cardiac pathologies such as Brugada syndrome and is involved in transient outward potassium current in the human heart [72,73].

Further studies in flies identified additional channel-encoding genes including *ether-a-go-go* (*eag*) [74], *eag*-*related gene* (*erg*), also named *seizure* (*sei*) and *eag-like K^+^ channel* (*elk*), known in vertebrates as *K_v_10, Kv11* and *Kv12*, respectively [75,76]. For instance, *eag* mutant flies exhibit slightly affected rhythmicity of the heart [44]. In humans, *HERG*, the ortholog of the *Drosophila erg* channel, caused arrhythmias (Long QT interval) [77]. Moreover, the first Ca^2+^ and voltage-dependent K^+^ channel *slowpoke* (*slo*) has been identified in *Drosophila* [78], with mouse and human *slo* genes cloned afterwards [79]. In mammals, abnormal intracellular Ca^2+^ handling and increased blood pressure have been related to mutations in the β1 subunit of *slo*, suggesting the *slo* channel activators could represent a good target of treatment in coronary heart disease [80,81].

In humans, the cardiac action potential repolarization depends on K^+^ channel currents categorized in early activating/inactivating transient outward currents (I_to_) and delayed rectifiers (I_k_) contributing to the later phase of membrane repolarization [82]. Multiple types of myocardial K^+^ channels contribute to the action potential waveforms and to the normal cardiac rhythm [83]. Mutations in these K^+^ channels lead to prolonged ventricular repolarization (*i.e.*, a prolonged QT interval), as manifested by Long QT syndrome, and ventricular tachycardia called *torsades des pointes*, life-threatening cardiac arrhythmias [84]. Most commonly, the delayed repolarization is due to mutations in the α-subunit of ion channels involving either *KCNQ1* (I_ks_) or *HERG* (I_kr_), responsible for the slow and rapid repolarizing of cardiac potassium currents [85,86]. Remarkably, the *Drosophila KCNQ* gene shares a conserved function with its human ortholog and maintains a rhythmic heartbeat. Null mutations in the fly *KCNQ* gene lead to repolarization defects characterized by prolonged contractions and increased arrhythmias, which worsen with age [48] (Table 1). In flies, the administration of KCNQ inhibitors, such as chromanol, phenocopies *KCNQ* mutant cardiac defects [87].

Interestingly, the young flies show a myogenic rhythmic beating pattern that deteriorates as they age, and by five to seven weeks *wildtype* flies exhibit non-rhythmic heart contractions with frequent asystoles and fibrillations [48]. The pronounced arrhythmias in aged flies are reminiscent of the age-dependent increase in atrial fibrillation in the aging human population [88]. Of note, cardiac-specific over-expression of *KCNQ* in aging *wildtype Drosophila* hearts strikingly reduced the incidence of arrhythmias [89]. Unlike the human heart, the K^+^ channels *HERG* and *KCNQ* do not play a role in action potential repolarization in adult murine hearts, with rapid repolarization and no clear plateau phase [90]. This suggests that the fly heart model may be a useful alternative for studying the K^+^ channel functions in cardiac repolarization and arrhythmogenic disorders.

In addition, the function of the two-pore domain potassium channel *ORK1* has been analyzed in *Drosophila*. *ORK1* mutants were found to display increased heart rate whereas over-expression of ORK1 can stop the heart from beating [40]. Also, the K^+^ channel named *dSUR* (*KATP*), known to be ATP-sensitive, was found to protect against hypoxic stress and pacing stress and, when mutated, induced heart failure in *Drosophila* [53]. The protective role of KATP has been observed in mammalian hearts under ischemia/reperfusion injury [91]. Pharmacological activation of the *KATP* channel in old flies by treating them with pinacidil reduced their susceptibility to pacing-induced heart failure [53], indicating that KATP channel activity contributes to a youthful heart performance (Table 1). In relation to ion channels, the *Drosophila* model has also been used to study atrial fibrillation disease, the most common clinical tachycardia, characterized by changes in electrical, structural and functional properties of cardiomyocytes [92]. Tachypacing *Drosophila* pupae resulted in a significant reduction in the amplitude of heart wall shortening and in contraction rate, and an increase in arrhythmias. Furthermore, the authors showed that genetic or pharmacological induction of heat shock proteins, especially *DmHSP23* (ortholog of human *HSPB1*), protects against tachypacing-induced contractile dysfunction [92]. Similar findings are observed in an *in vitro* atrial cell line model of tachycardia and *in vivo* in dogs subjected to atrial fibrillation [93].

### 2.2. Cardiomyopathies

In mammals, as in *Drosophila*, mutations in sarcomeric or cytoskeletal/sub-membranous proteins have been implicated in the pathogenesis of the inherited cardiomyopathies.

#### 2.2.1. HCM

HCM is a relatively common genetic disease caused by a variety of gene mutations, the majority of which encode sarcomere proteins [94]. Several hundred distinct mutations in over a dozen proteins have been identified in patients with HCM [95]. These include cardiac myosin binding protein C (MYBPC3), cardiac α-myosin heavy chain (MYH6), cardiac β-myosin heavy chain (MYH7), cardiac troponin T (TNNT), cardiac troponin I (TNNI3), cardiac troponin C (TNNC1), regulatory and essential myosin light chain (MYL2 and MYL3), α-tropomyosin (TPM1), titin (TTN), and cardiac actin (ACTC1) [96,97]. Other proteins account for few cases of HCM and include myozenin 2 (MYOZ2), MLP (CSRP3), telethonin (TCAP), metavinculin (VCL) and junctophilin-2 (JPH2) and so forth [98,99,100,101,102]. Data from human patients suggest that approximately 60% of HCMs occur from dominant mutations of sarcomere protein genes and, among those, *MYH7* and *MYBPC3* predominate in frequency [103,104]. For example, several reports on the molecular performance of myosin revealed compromised motor biomechanical functions and, hence, hypo-contractile myocardium which is compensated by hypertrophy [105,106]. However, other studies show that specific HCM mutations isolated from murine cardiac tissue encode myosin with enhanced functional properties [107,108], suggesting that the pathophysiology of HCM is quite variable and diverse related to particular mutations, causal mechanisms and the hypertrophic response.

Recently, it has been shown that the *Drosophila* heart can undergo hypertrophy, similar to humans, in response to signals of receptor tyrosine kinase EGFR (Epidermal Growth Factor Receptor) and the downstream molecules such as the small GTP-ase Ras and the serine/threonine-specific protein kinase Raf [57] (Table 1). The activation of EGFR, Ras and Raf increases the heart wall thickness and reduces the cardiac lumen size at diastole in adult flies. Heart-specific down-regulation of extracellular signal-regulated kinase (ERK), one of the downstream effectors of Raf, prevented Raf-mediated cardiac hypertrophy. Moreover, it has been shown that Yorkie (Yki), a transcriptional coactivator in the Hippo pathway, also is stimulated by Raf to promote cardiac hypertrophy and the cardiac-specific knockdown of Yki inhibited Raf-mediated HCM in flies [59]. The authors showed in flies as well as in mammalian cells that Yki induces activity of a common downstream transcription factor, Scalloped. In addition, expressing constitutively active calcineurin, a calcium/calmodulin-dependent protein phosphatase, in the fly heart induced cardiac hypertrophy, similar to mammals where calcineurin is a known mediator of cardiac hypertrophy [60,109,110,111]. Interestingly, a genetic screen for modifiers of the calcineurin-mediated hypertrophy in flies identified galactokinase as a new suppressor of the cardiac remodeling [60]. It remains now to verify if this regulator is involved in the HCM pathway in mammals (Table 1).

#### 2.2.2. DCM

DCM is caused by mutations in various genes encoding sarcomeric proteins, cytoskeletal proteins, sarcolemmal membrane and nuclear envelope proteins [112,113,114]. These causative genes include *dystrophin*, *desmin*, *lamin A/C*, δ*-sarcoglycan*, β*-sarcoglycan*, *metavinculin*, β*-myosin heavy chain*, *myosin-binding protein C*, *actin*, α*-tropomyosin*, *cardiac troponin T* and *C*, *telethonin*, *phospholamban* and sodium-channel gene *SCN5A*, and so forth [115]. It is important to note that the HCM-causing mutations in many sarcomeric proteins are different from those inducing DCM pathogenesis. For example, the DCM-causing mutations in TNNT2 decrease the myofilament sensitivity to cytoplasmic Ca^2+^ and thus impair the systolic function [116,117,118,119]. The only TNNT mutation tested in flies so far is Glu88Lys, the one that may compromise the tropomyosin-TNNT associations and shows diastolic dysfunction, a hallmark of HCM and RCM [58]. It remains to be studied if this TNNT mutation tested in flies has decreased the calcium-dependent regulatory role in myofilament activation.

Interestingly, functionally depressed *MHC* mutant fly hearts characterized by a decreased actin-sliding velocity are significantly dilated and exhibit systolic dysfunction reflected by a drop in fractional shortening [55]. Of note, *Unc-45* knockdown, a chaperone protein necessary for myosin folding, also displays DCM [120]. Likewise, in mammals, *MHC* mutations found to be associated with DCM have been implicated in the disruption of myosin-actin interactions, possibly decreasing the actomyosin ATPase rates [108,121]. Other sarcomeric mutations showed a similar effect on the *Drosophila* heart tube. Mutations in Troponin I and Tropomyosin 2, which cause aberrant contractile properties, also show enlarged hearts, depressed fractional shortening and cause a phenotype reminiscent of DCM [50] (Table 1).

**Figure 2:**
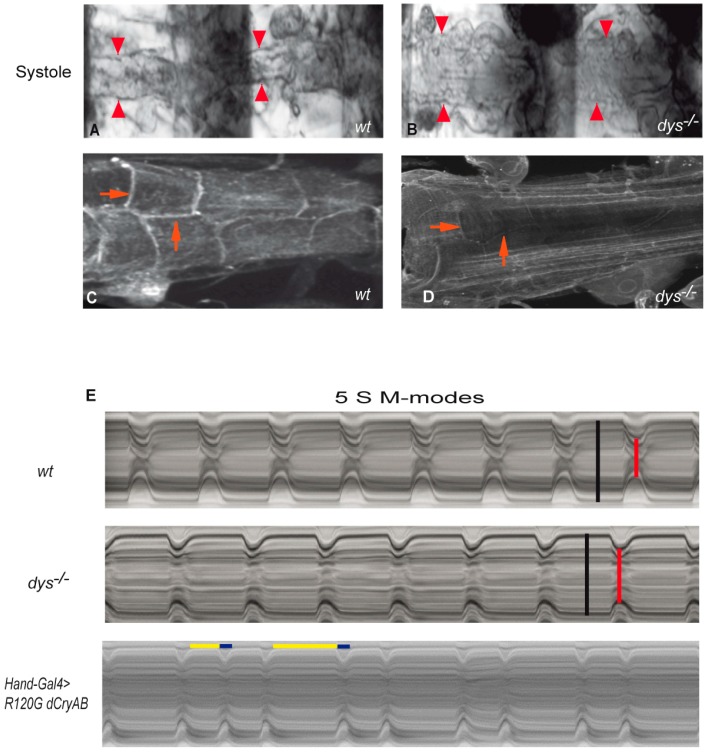
Examples of phenotypes reminiscent of dilated cardiomyopathy and arrhythmia in fly hearts. (**A**) Image of two abdominal segments of a one-week-old *wildtype* (*wt*) heart in systole; (**B**) One-week-old *dystrophin* (*dys*^−/−^) mutant heart in systole showing a dilated phenotype. Note that the systolic diameters are wider in the *dys*^−/−^ mutants compared to *wt*. Arrowheads indicate the heart wall in both genotypes; (**C**,**D**) Representative confocal stacks of a segment of adult hearts stained with Dg antibody. Dg is found at the cell membrane of the cardiomyocytes (arrows); (**E**) Representative M-mode traces (5 s) illustrating movements of heart tube walls (*Y*-axis) over time (*X*-axis). Diastolic (**black**) and systolic (**red**) diameters were indicated in each M-mode trace. Wild-type flies show rhythmic heart beating at one-week-old and smaller systolic diameters, compared to *dy*-deficient heart. Diastolic (**yellow**) and systolic (**blue**) intervals were indicated above the *Hand-Gal4* > *R120G dCryAB* M-mode trace. Note the expression of a mutation of the small heat shock protein CryAB (R120GdCryAB) induces arrhythmia in flies. Please replace.

Examples of cytoskeletal/sub-membranous protein mutations have been associated with DCM. Transgenic flies harboring the cardiac-specific expression of human δ-sarcoglycan^S151A^, a mutation associated with familial DCM, demonstrated enlarged cardiac tubes and impaired systolic function in comparison to the expression of the human *wildtype* allele [50]. Likewise, fly mutants lacking the δ*-sarcoglycan* gene developed DCM [122]. As in mammals, *Drosophila* recapitulates cardiac phenotypes caused by *dystrophin* mutations. Interestingly, mutant flies for *dystrophin* developed distended systole and diastole diameters, impaired systolic function and age-dependent abnormalities in heart myofibrillar organization [56] (Table 1) (Figure 2).

As for cytoarchitectural proteins, fly mutants of signaling pathway components such as rhomboid 3 (rho-3), an intramembrane serine protease involved in EGF activation, as well as the cardiac-specific inhibition of the EGFR cause an enlarged cardiac chamber [123]. Similarly, affecting the EGFR function in humans by chemotherapies induces the development of DCM and heart failure [124]. Screening flies harboring genomic deficiencies identified a novel Notch ligand named weary (wry) that induces DCM, suggesting that Notch signaling is important for normal heart function [125]. In addition, an *in vivo* RNAi adult heart screen in *Drosophila* identifies Not3, a component of the CCR4-Not complex. Silencing CCR4-Not components in flies resulted in DCM and myofibrillar disarray, and subsequent experiments in mice show that Not3 haploinsufficiency resulted in impaired cardiac contractility [126]. Thanks to this data, a single-nucleotide polymorphism in the human NOT3 promoter that is associated with prolonged QT intervals and sudden death has been identified [126] (Table 1).

Recent work from the Bodmer laboratory showed that the small Rho-GTPase encoded by *Cdc42* interacts with *tin* to regulate heart function in flies. Double heterozygous mutants for *tin*/*Cdc42* turned out to have a slower heart rate and increased arrhythmias, and showed that the two K^+^ channels *dSUR* and *slo* act as downstream mediators of the tin-Cdc42 pathway [29]. Interestingly, haploinsufficiency of mouse *Cdc42* and *Nkx2-5* resulted in dilated and defective cardiac contraction, suggesting a conserved genetic and functional Cdc42/*Nkx2-5* interaction in mammals [29].

#### 2.2.3. RCM

RCM is characterized by decreased volumes of both ventricles and pronounced diastolic dysfunction which results from decreased myocardial wall elasticity. To date, RCM-linked mutations are found in sarcomere protein genes, including *ACTC1*, *TNNI3*, *TNNT*, *MYH7* [127,128,129]. In addition, missense variants in the *desmin* gene have been identified in several families with desmin-related myopathy and presenting RCM [130]. *In vitro* analysis revealed that the RCM-causing sarcomeric gene mutations drastically increase the myofilament sensitivity to cytoplasmic Ca^2+^, a greater enhancement compared to the HCM mutations [131].

In contrast to the cardiac phenotype resulting from depressed myosin function, *Drosophila* hearts expressing kinetically and mechanically enhanced myosin motors displayed morphological and functional cardiac characteristics associated with RCM [55]. The cardiac tubes expressing the hyperactive Mhc^5^ myosin exhibit a narrowing of the heart chamber and impaired diastolic function, a phenotype which progressively worsened with age. In addition, *Drosophila* expressing glutamic acid to lysine *Up*^101^ troponin-T mutation exhibit a restrictive and stiffer phenotype with diastolic dysfunction, a variant causing elevated numbers of basally cycling myosin cross-bridges [58] (Table 1).

## 3. Pathological Mechanisms Investigated

### 3.1. Impaired Calcium Handling

Cardiac calcium signaling is an important factor in regulating excitation-contraction coupling for proper contraction and relaxation of the heart [132]. The amount of extra- and intra-cellular Ca^2+^ must be tightly controlled by several genes encoding L- and T-type voltage-gated Ca^2+^ channels, Na^+^/Ca^2+^ exchangers (NCX), dihydropyridine receptors, ryanodine receptor (RyR), SR Ca^2+^ ATP-dependent carrier (SERCA) and phospholamban (a SERCA inhibitor). Mutations in one of these proteins perturb Ca^2+^ homeostasis and contribute to diastolic dysfunction, causing heart failure or heart hypertrophy [133,134]. The SERCA mutant studies in the fruitfly showed reduced heartbeat as well as increased arrhythmia [135,136]. Also, the SERCA-interacting protein *sarcolamban* (*scl*), named after its mammalian orthologs *phospholamban* (*pln*) and *sarcolipin* (*sln*), has been involved in heart physiology since *scl* mutants exhibit arrhythmic heart contractions that can be linked to Ca^2+^ transport deficit [137].

Lin and colleagues measured myocardial Ca^2+^ transients in adult flies using cardiac-specific, genetically encoded, calcium-dependent fluorescent protein (GCaMP2). The authors showed, in troponin I mutants that exhibit impaired cardiac function and dilated cardiomyopathy, abnormalities in cardiac calcium handling, reduced expression of ryanodine receptor transcripts and a decreased response to caffeine-augmented calcium release [138]. Of note, the reduction in the RyR transcript level is consistent with the prolonged duration of 50% Ca^2+^ rise to peak intensity in the cytoplasm [138]. In addition, *Ryr*^16^ larval mutants exhibit a reduced heart rate, mainly due to decreased fast contractions, attesting the functional conservation of RyR with vertebrates [139]. Moreover, as described above, *sarcolamban* mutants exhibit altered rhythmic contractions due to perturbed calcium current and lead to delayed or absent action potential [137], indicating that many genes involved in Ca^2+^ handling within cardiomyocytes are functionally conserved between *Drosophila* and mammals.

### 3.2. Altered Metabolism

The heart is the organ with the most important demand in energy within the whole body, due to high consumption caused by the excitation-contraction coupling process. Adenosine Triphosphate (ATP) generated from its main substrates, fatty acids, ketone bodies and carbohydrates provides this energy source [140,141]. The balance between energy demand and consumption needs to be controlled for efficient cardiac contraction-relaxation cycles because of the low ATP reserve in the heart. When energy uptake is increased, such as in Western countries due to high fat and high sugar diets, it leads to increased triglyceride and glucose levels, causing obesity and diabetes associated with heart dysfunction. Metabolic syndrome caused by a high fat diet is a disorder recapitulating these metabolic perturbations (obesity and increased risk of type 2 diabetes mellitus) with increased incidence of cardiomyopathy [142]. Due to the conservation of fat and sugar metabolisms in *Drosophila*, this model has been used to uncover the pathways deregulated in these contexts [138,143,144,145]. For instance, Birse and colleagues showed that feeding the flies a high fat diet caused a metabolic syndrome similar to what is observed in humans with cardiac dysfunction involving the Insulin-TOR pathways. The high calorie diets induced an increase in Insulin-like peptide 2 (dIlp-2) transcript levels as well as activation of the TOR pathway, as measured by increased 4EBP and S6K phosphorylation [143]. Moreover, a high sugar diet (HSD) has also been assessed in flies and caused insulin resistance and type 2 diabetes with the development of cardiomyopathies. Flies fed with HSD have a reduced lifespan together with increased arrhythmia and deterioration of the heart which are modulated by the Insulin and MAPK pathways [146]. Since patients with a HSD tend to develop diabetes usually associated with cardiac fibrosis, Na and colleagues assessed the level of extracellular matrix proteins in HSD-fed flies [146]. They showed an increase of the type IV collagen–like protein Pericardin, demonstrating the fibrosis-like collagen accumulation in flies with other signs of diabetic cardiomyopathy, reminiscent of the symptoms observed in type 2 diabetes mellitus patients [146]. More recently, the key metabolic genes modulating cardiac lipotoxicity were described, showing the protective role of *Drosophila* Peroxisome proliferator-activated receptor γ coactivator-1 (PGC1), called *spargel* (*srl*), downstream of the TOR pathway [147]. The deleterious high calorie diets–induced cardiac dysfunction is alleviated by down-regulating brummer, the fly homolog of adipocyte triglyceride lipase (ATGL), and spargel, suggesting that cardiac-specific inhibition of TOR blocks the heart effects of a high caloric diet in flies [143,146,147]. Likewise, the induction of SREBP by TOR activation, in parallel to ATGL inhibition, caused FAS induction and lipid storage. These studies describe the genetic network involving several metabolic regulators of lipotoxic cardiomyopathy including the Insulin-TOR pathway, ATGL, PGC1 and SREBP orthologs, already described for their implication in obesity in humans [148]. More recently, the effect of time-restricted feeding (TRF) was studied in the fly, showing its protective effect against age-related cardiac decline compared to a normal unlimited diet [149]. Five-week-old flies fed in a 12 h time-restricted manner have improved cardiac function shown by shorter heart periods, less arrhythmia and better fractional shortening, which has been correlated to the increased sleep duration of these TRF flies. Importantly, TRF protects against the cardiac decline caused by a high fat diet and is mediated by the ATP-dependent TCP-1 ring complex (TRiC), the mitochondrial electron transport chain complex, as well as the circadian clock pathways. In mice, TRF was already demonstrated to be a non-pharmacological lifestyle strategy to prevent obesity and its associated-metabolic deregulations [150] since TRF reduces body weight gain and improves hepathic glucose metabolism in mice fed either with a normal or a high fat diet [150]. Of note, the TRF regimen protected against the perturbation of metabolic regulators such as mTOR and AMPK in comparison to mice fed a high fat diet frequently throughout the day and night.

Moreover, the role of cardiac aging in metabolism regulation has been extensively studied in *Drosophila*. Aging flies exhibit altered heart metabolism with increased arrhythmia, myofibrillar disorganization and dysregulation of Insulin and TOR pathways. Strikingly, reducing Insulin or TOR pathway activation increases lifespan and delays cardiac aging [151,152,153], showing that aging can be controlled by nutrient-sensing (reviewed elsewhere [154]).

### 3.3. Increased Oxidative Stress and Mitochondrial Dysfunction

Oxidative stress caused by reactive oxygen species (ROS), hydrogen peroxide (H_2_O_2_) or superoxide anions (O_2_^−^) has deleterious effects on cells and can cause damage. In flies, significant elevation or reduction of ROS causes cardiac dysfunction [155]. The authors showed that manipulating the ROS in pericardial cells (PCs) could regulate the heart function in a paracrine manner. ROS activate a p38MAP kinase–dependent signaling cascade in PCs, which affects myocardial cell function and this mechanism does not act through the diffusion of ROS [155]. The Huntington disease fly model, in which the accumulation of polyglutamine (poly-Q) aggregates increases oxidative stress, causes cardiac defects such as increased arrhythmia and cardiac dilation [156]. The authors demonstrated an increase in dihydroethidium (DHE), reflecting ROS production in cells, after inducing poly-Q repeats in cardiac tissue as well as an increase in density aggregates in mutant flies fed with H_2_O_2_. However, the over-expression of superoxide dismutase (SOD), an antioxidant enzyme which catalyzes O_2_^-^ detoxification, rescued the poly-Q–induced cardiomyopathy. In addition, the over-expression of the antioxidant enzyme catalase suppresses age-induced arrhythmias [157].

Increased reductive stress can also be toxic for the cell, as shown in the Desmin-related myopathy (DRM) *Drosophila* model, using human α*B-crystallin* mutant flies (expressing the human *CryAB^R120G^* mutation) that exhibit similar cardiac symptoms as those observed in patients. This model showed a mitochondrial NADP/H metabolism implication in increasing reductive stress and affecting the heart function [158,159]. In more detail, the human *CryAB^R120G^* mutation expressed in flies leads to increased diastolic and systolic diameters, decreased fractional shortening, as well as arrhythmias (Figure 2E), which is reminiscent of DCM in patients. These cardiac defects were alleviated by a cardiac-specific knockdown of glucose-6-phosphate dehydrogenase (G6PD), an enzyme involved in the generation of NADPH as well as its downstream effector, 6-phosphogluconate dehydrogenase (PGD).

### 3.4. Remodeling of Extracellular Matrix

Among the extracellular matrix (ECM) proteins, fibrillar proteins such as collagen and proteoglycans may play a role in determining the properties of the myocardium [160]. The fly has been used for fine genetic mapping of candidate genes involved in human congenital heart diseases with Down syndrome. Over-expression of these candidate genes individually or in pairwise combinations in the adult fly heart identified DSCAM and COL6A2 as the most strongly interacting pair of genes, causing a slower and less rhythmic heart rate. Afterwards, the same combination of genes has been expressed in the mouse heart, leading to morphological and physiological defects [161]. Double-transgenic mice exhibit increased heart wall thickness of the left ventricle and the interventricular septum (hypertrophy phenotype). Several transcripts were affected in the hearts of the double-transgenic mice over-expressing DSCAM and COL6A2, including those encoding focal adhesion protein Tenascin N, cardiac Troponin T and calcium-binding protein S100A4. Further analyses of identified candidate genes in the *Drosophila* model will help in understanding the impact of extracellular matrix remodeling on cardiac function.

## 4. Challenges in CVDs Modeling

Examples provided here clearly show that the *Drosophila* heart model has already proved its value in investigating regulatory pathways that underlie CVDs. Numerous factors that are conserved between flies and humans correspond to cytoarchitectural or signaling components bearing pathological mutations known to cause CVDs. The ease in using *Drosophila* genetics makes it possible to design screens to identify second-site modifying mutations such as enhancers and suppressors of mutant phenotypes. Similar genetic interaction screens in vertebrate models are problematic because of time, cost, and early lethality or modest mutant phenotypes due to genetic redundancy. Of note, the lack of genetic redundancy in flies may represent a limitation since the associated gene regulatory networks that are present in the vertebrate system may not be functional in the fly. Also, because of evolutionary distance and morphological differences, *Drosophila* cannot be an exact model of detailed heart physiology. The fly heart has a single chamber and is a part of an open circulatory system with anterograde and retrograde beats controlled by two pacemakers [42,162]. These reversal beats can cause irregularities in the adult heart rate, making assessment of the conduction defects difficult in intact flies. The *Drosophila* heart is also not adapted to perform ischemia/reperfusion studies as it has a single layer of cardiomyocytes without coronary arteries and relies on oxygen delivery by diffusion. However, a short lifecycle and conserved genetic networks governing cardiac development and function make this organism an attractive model for investigating the inherited CVDs, including cardiomyopathies and channelopathies. In addition, several genetic tools have been developed in flies during recent years that have further strengthened this model and permit unparalleled investigative power relative to other model systems. It is now possible to manipulate different binary expression systems to independently target cell type, level and timing. In such strategies, gene over-expression, misexpression or silencing due to RNA interference or dominant-negative proteins can be achieved using the bipartite GAL4-UAS system (GAL4-Upstream Activating sequence), the LexA system, the split Gal4/LexA system, and the Q system [163,164,165,166,167]. In conjunction with the large collections of fly mutants and cardiac phenotyping techniques available, the *Drosophila* heart continues to be efficient for uncovering new modifier genes that affect the penetrance of the phenotypes and for revealing polygenic interactions that are difficult to pursue in the mammalian heart.

One of the major challenges is the large number of DNA sequence variants, generated by the new sequencing technologies and personalized medicine. Distinguishing the pathogenic mutations from the background of human genetic polymorphism has emerged as problematic for interpreting the genetic test results of the cardiomyopathy/channelopathy diagnosis [168,169,170]. Owing to the complexity involved in correctly interpreting DNA variants, and in order to differentiate pathogenic disease-causing mutations from otherwise polymorphism, *in vivo* testing in fly heart function could be informative and can be complementary to the variant interpretation algorithms, which are under development.

Interestingly, the *Drosophila* heart has been used to easily assess the potential effect of drug therapy. For example, drug screening allowed the identification of candidate drugs for the treatment of atrial fibrillation (AF) in the fly. Tachypacing of fly pupae resulted in cardiomyocyte remodeling, reduced the contraction rate, and increased arrhythmia and reduction in heart wall shortening. A preventive protective effect is observed in tachypaced pupae after a heat-shock or pretreatment with HSP-inducers GGA and BGP-15 [92,171]. Similarly, HDAC and Sirtuins have also been implicated in AF since the treatment with HDAC or Sirtuin inhibitors (Tubacin and Nicotinamide, respectively) before tachypacing induction prevents the *Drosophila* heart from contractile dysfunction, which is reminiscent of human AF [172].

Moreover, drugs can be of help in validating the role of several ionic channels by using specific inhibitors of these channels and analyzing the fly heart physiology. For example, the administration of K_ATP_ channel inhibitor tolbutamide in flies phenocopied the increased heart failure rate observed in *dSUR* mutants. In the same way, the chromanol *KCNQ* inhibitor mimicked the increased arrhythmicity observed in *KCNQ* mutants [87].

More recently, work from Chakraborty and colleagues tested the role of Pentamidine, a drug previously involved in reducing toxic foci in myotonic dystrophy type 1 (DM1) cultured cells and mice models [173]. They showed decreased cardiac arrhythmia and improved contractility that could be due to muscleblind release from nuclear foci in adult flies expressing 250 CUG specifically in the heart [174]. Moreover, methylene blue (MB) also showed its beneficial effect on two disease models with heart involvement, Friedreich’s ataxia (FRDA) and Huntington disease (HD). In FRDA, MB rescued the heart dilation caused by heart-specific frataxin depletion whereas MB partially protected the *Drosophila* heart against mutant huntingtin expression due to improvement of mitochondrial function [175,176].

Recent advances in genetic and imaging tools available to study heart function in flies open a possibility to apply this powerful model for high-throughput drug screenings and for the preclinical evaluation of drug effects. So far, cell-based assays or isolated tissues are used for the selection of cardiovascular-safe drugs, where physiological effects cannot be monitored. Thus, the fly model, by providing a mean to evaluate the drug-induced changes in cardiac electrophysiology and in the structural integrity of heart tissue, appears as an attractive, complementary method for cardiac drug screening and assessment.

## 5. Conclusions

While the fruitfly heart is a simple linear cardiac tube in comparison with higher vertebrates, investigations of the *Drosophila* heart have yielded new insights into mammalian cardiac development and function. The morphological differences that exist between the fly and the human heart (e.g., single chamber, lack of coronary circulation and non-cardiac cells) limit the utility of the fly as a model for certain types of human heart disorders such as coronary diseases. Nevertheless, the above findings suggest that the molecular pathways involved in the inherited cardiomyopathies and the channelopathies may be conserved between flies and humans since many cytoarchitectural and signaling protein mutations cause similar heart defects in flies.

Identification of molecular and functional mechanisms of heart disease–causing mutations is essential to understand the pathways that link an altered gene to a clinical phenotype. The development of transgenic models such as flies has been proven to be efficient for functional studies in cardiovascular diseases. In fact, *Drosophila* genetics provide a mean to (1) identify genes and pathways that potentially contribute to channelopathies and cardiomyopathies; (2) discover the mechanisms by which protein mutations trigger signals that result in the remodeling of the heart; (3) test, verify or validate the pathogenic DNA variants associated with heart diseases; and (4) screen for pharmacological agents to identify novel therapeutics. Given the range of heterogeneity and phenotypes of the familial cardiomyopathies and channelopathies, the role of modifier screens in flies will be beneficial in gaining time for identifying new components or mechanisms involved in human cardiovascular diseases.

## Figures and Tables

**Table 1:** *Drosophila* models of channelopathies and cardiomyopathies.

Disease Type		Human Gene	*Drosophila* Model	Major Findings in the *Drosophila* Model	References
**Channelopathies**	**K^+^ channels**	*KCNQ1*	*KCNQ* mutants	Increased arrhythmia and prolonged contractions	[48]
		*KCNK*	*ORK1* mutants	Increased HR	[40]
		*SUR2*	*dSUR* mutants	Heart failure Protective role against hypoxia and pacing-stress	[53]
		*KV2.1*	*Shab* mutants	Reduced HR	[54]
**Cardiomyopathies**	**Hypertrophic CM**	*EGFR, RAS, RAF1*	Cardiac-specific expression of *EGFR*, *Ras*, *Raf* or *Yki*	Increased heart wall thickness Decreased cardiac lumen volume	[57,59]
		*Calcineurin*	Cardiac-specific expression of constitutively activated *CanA*	Enlarged DD and reduced FS Heart wall thickening Identification of *Galk* as a repressor of *CanA* induced HCM	[60]
	**Dilated CM**	*TNNI3 and TPM1*	*wupA* and *Tm1* mutants	Enlarged diameters and impaired systolic function	[50]
		*DMD*	*Dys mutants*	Dilated DD, SD and reduced FS DCM rescue by expressing mammalian form of *Dys* (*Dp116*)	[56]
		*SGCG*	Human δ*-Scg*^S151A^/Large deletion of *Scg*δ	Enlarged heart tube, impaired systolic function and reduced FS	[50,122]
		*Myosin Heavy Chain (MYH7)*	Hypoactive *D45 Mhc* mutants	Dilated heart and decreased FS	[55]
		*RHBDL2*	*rho-3* mutants	Enlarged cardiac chamber rescued by expression of *rho-3*, *spitz* and *EGFR*	[123]
		N/A	Cardiac-specific *Wry* RNAi and mutant	Enlarged DD/SD and decreased FS in *wry* mutants rescued by *Notch* expression Identification of *Wry* as a *Notch* ligand	[125]
		*CCR4-NOT3*	Cardiac-specific knockdown of *Not3*	Increased DD, SD and reduced FS	[126]
	**Restrictive CM**	*Myosin Heavy Chain (MYH7)*	Hyperactive *Mhc^5^* mutants	Impaired diastolic function and restrictive heart phenotypes	[55]
		*TNNT2*	*Up^101^* mutants	Reduced DD, SD and FS Prolonged periods of systole and reduced HP	[58]

Abbreviations: HR: heart rate; HP: heart period; SD: systolic diameter; DD: diastolic diameter; FS: fractional shortening, N/A: not applicable (No known ortholog).
